# Interplay between FLI-1 and the LDB1 complex in murine erythroleukemia cells and during megakaryopoiesis

**DOI:** 10.1016/j.isci.2021.102210

**Published:** 2021-02-20

**Authors:** Guillaume Giraud, Petros Kolovos, Ilias Boltsis, Jente van Staalduinen, Boris Guyot, Michele Weiss-Gayet, Wilfred van IJcken, François Morlé, Frank Grosveld

**Affiliations:** 1 Department of Cell Biology, Erasmus Medical Centre, 3015CN Rotterdam, the Netherlands; 2 Department of Molecular Biology and Genetics, Democritus University of Thrace, Alexandroupolis 68100, Greece; 3 CNRS UMR5286, Centre de Recherche en Cancérologie de Lyon, Lyon, France; 4 Institut NeuroMyoGène, CNRS UMR 5310 - INSERM U1217 - Université de Lyon - Université Claude Bernard Lyon 1, Lyon, France; 5 Biomics Center, Erasmus University Medical Center, 3015CN Rotterdam, the Netherlands; 6 Inserm U1052, Centre de Recherche en Cancérologie de Lyon, Lyon, France; 7 Université de Lyon, Lyon, France; 8 Department of Immunity, Virus and Microenvironment, Lyon, France

**Keywords:** Molecular Biology, Chromosome Organization

## Abstract

Transcription factors are key players in a broad range of cellular processes such as cell-fate decision. Understanding how they act to control these processes is of critical importance for therapy purposes. FLI-1 controls several hematopoietic lineage differentiation including megakaryopoiesis and erythropoiesis. Its aberrant expression is often observed in cancer and is associated with poor prognosis. We showed that FLI-1 interacts with the LDB1 complex, which also plays critical roles in erythropoiesis and megakaryopoiesis. In this study, we aimed to unravel how FLI-1 and the LDB1 complex act together in murine erythroleukemia cells and in megakaryocyte. Combining omics techniques, we show that FLI-1 enables the recruitment of the LDB1 complex to regulatory sequences of megakaryocytic genes and to enhancers. We show as well for the first time that FLI-1 is able to modulate the 3D chromatin organization by promoting chromatin looping between enhancers and promoters most likely through the LDB1 complex.

## Highlights

• FLI-1 is important for the recruitment of the LDB1 complex
• FLI-1 is important for chromatin looping
• FLI-1 and the LDB1 complex co-regulate megakaryopoiesis

## Graphical abstract

**Figure:**
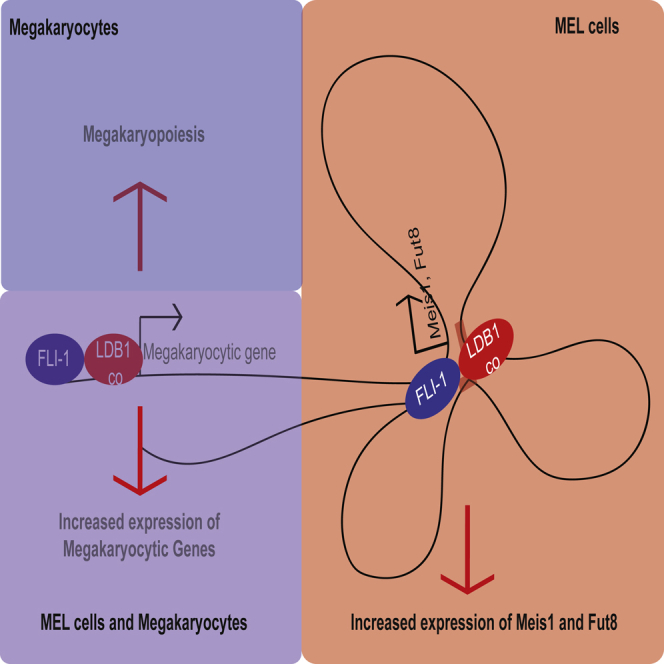

## Introduction

Transcription factors (TF) play critical roles in a broad range of cellular processes such as cell-fate decision and proliferation. They act as protein complexes to directly regulate gene expression through their recruitment to regulatory sequences. Their aberrant expression, which triggers perturbation in their molecular networks, is often the primary cause of cancer (Bhagwat and Vakoc, 2015; Nebert, 2002). Moreover, this class of proteins is also used as a cocktail of TF to reprogram differentiated cells into pluripotent stem cells (Rapino et al., 2013; Takahashi and Yamanaka, 2006). Therefore, understanding how TFs act to control these different processes remains critical to improve therapeutic strategies for cancer or for regenerative medicine.

Fli-1 TF belongs to the ETS family, whose members are characterized by a conserved ETS DNA-binding domain recognizing a purine-rich motif, GGAA (Wei et al., 2010). *Fli-1* was first identified as the main integration site of the Friend helper virus (F-MuLV), which triggers erythroleukemia in mice (Ben-David et al, 1990, 1991). In murine erythroleukemia (MEL) cells, Fli-1, together with another ETS TF, Pu.1, contributes to the proliferation, the survival, and the block of differentiation of erythroid progenitors (Juban et al., 2009). In humans, *Fli-1* aberrant expression is observed in autoimmune diseases as well as in hematopoietic and non-hematopoietic cancer and is often associated with poor prognosis (Kornblau et al., 2011; Suzuki et al., 2012; Yan et al., 2018). Next to this role in pathology, Fli-1 targeted mice display defects in several hematopoietic lineages such as granulocytes, erythrocytes, and megakaryocytes (Kawada et al., 2001; Masuya et al., 2005; Moussa et al., 2010; Spyropoulos et al., 2000). In particular, Fli-1 promotes megakaryopoiesis at the expense of erythropoiesis (Starck et al., 2010). Interestingly, Fli-1 has been used in combination with GATA1 and TAL1 TF to enhance megakaryocyte production from pluripotent stem cells, which has its importance in transfusion-based therapies (Moreau et al., 2016). Despite these well-established contributions of Fli-1 during physiological and pathological development, the molecular mechanisms by which this important TF acts still remain elusive.

GATA1 and TAL1 are part of a same protein complex, namely the LDB1 complex. This complex, which also contains the E2A TF and two bridge proteins, LDB1 and LMO2, act as a platform to recruit either co-activators or co-repressors to regulate gene expression (Love et al., 2014). The LDB1 complex is important at all the steps of erythropoiesis including the expansion of erythroid progenitors and the terminal differentiation. To do so, the LDB1 complex is mainly recruited to enhancers of critical genes involved in these processes such as *c-Myb* or *ß-globin* and promotes chromatin looping to place these enhancers in close proximity to the targeted promoter to activate their expression (Krivega et al., 2014; Krivega and Dean, 2017; Lee et al., 2017; Li et al., 2013; Stadhouders et al, 2012, 2014; Soler et al., 2010). Next to this critical role during erythropoiesis, the LDB1 complex is also important for megakaryopoiesis (Hamlett et al., 2008). However, how it works in this context is not fully described yet.

We have previously shown that Fli-1 and the LDB1 complex interact in MEL cells (Giraud et al., 2014). We hypothesized that Fli-1 and the LDB1 complex control the expression of common target genes. We used MEL cells as a cellular model to decipher how Fli-1 works in combination with the LDB1 complex. By using omics techniques, we show that Fli-1 and the LDB1 complex are mainly recruited to active enhancers where Fli-1 enables the recruitment of the LDB1 complex and their chromatin looping to the corresponding promoter, which demonstrates for the first time a role of FLI-1 in the 3D structure of the genome. We also show that in MEL cells as in megakaryocytes, Fli-1 and the LDB1 complex directly activates the expression of megakaryocytic genes and that they cooperatively regulate megakaryopoiesis.

## Results

### Fli-1 binds active regions containing the ETS and the TAL1:GATA1 motifs

**Figure 1:**
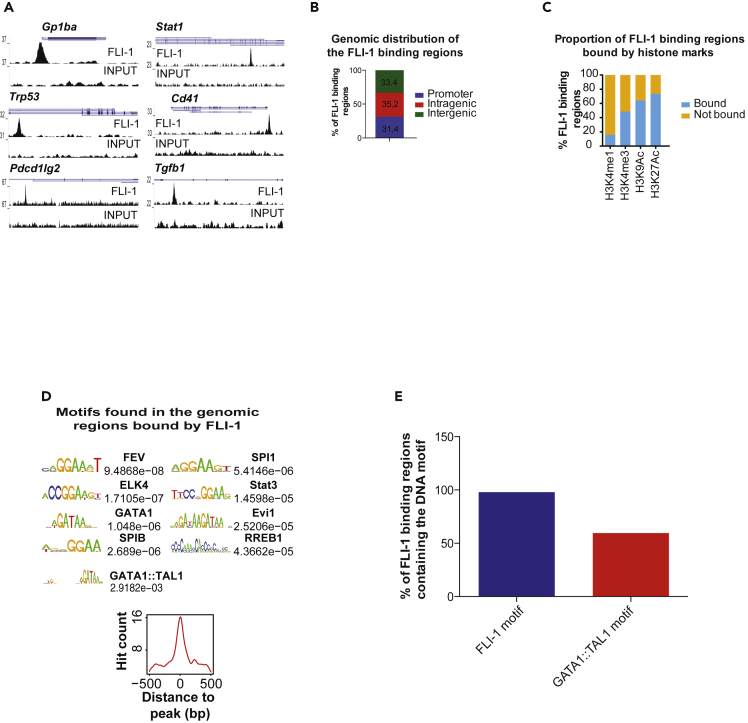
Fli-1 binds active regions containing the ETS and the TAL1:GATA1 motif in MEL cells (A) Binding sites of Fli-1 observed in the Gp1ba, Stat1, Trp53, Cd41, Pdcd1lg2, and Tgfb1 loci in MEL cells. (B) Percentage of Fli-1 binding regions in MEL cells located to promoter (1 kb upstream and downstream of the TSS, blue bars), intragenic (red bars), and intergenic (green bars) regions. (C) Percentage of Fli-1 binding regions bound (blue bars) or not bound (yellow bars) by H3K4me1, H3K4me3, H3K9Ac, or H3K27Ac in MEL cells. (D) List of the motifs enriched in the regions bound by Fli-1 in MEL cells (left panels) and their respective centrality compared with the FLI-1 peak (bottom panels). (E) Proportion of Fli-1 bound regions containing the Fli-1 (blue bar) or the GATA1:TAL1 motif. See also Figure S1 and Table S3.

To unravel the mode of action of FLI-1, we first performed Fli-1 ChIP-Seq in MEL cells. With a comprehensive bioinformatical analysis, we identified 1,116 Fli-1 genome-wide bound regions and verified them by ChIP-qPCR assays in non-induced and DMSO-induced MEL cells (Figures 1A and S1A). As expected, the depletion of Fli-1 by shRNA in both states decreases the signal observed in these regions highlighting its specificity (Figures S1A–S1C). Fli-1 binding regions are preferentially located at gene bodies (Figure 1B) and the majority is in regions marked by the H3K9Ac and H3K27Ac histone modifications, corresponding to active chromatin regions (Figure 1C). We identified the DNA-binding motifs in the vicinity of the Fli-1 bound regions and as expected based on previous studies, more than 95% of the Fli-1 binding regions are enriched for the ETS motif (Figures 1D and 1E) (Wei et al., 2010; Wilson et al., 2010). Strikingly, almost 60% of these Fli-1 binding regions have the TAL1:GATA1 motif (Love et al., 2014; Soler et al., 2010), which recruits the LDB1 complex suggesting that FLI-1 is recruited together with the LDB1 complex in its target regions (Figures 1D and 1E).

Taken together, these analyses show that Fli-1 binds active regions containing the ETS motif and the TAL1:GATA1 motif.

### Fli-1 and the LDB1 complex are mainly bound to active enhancers in MEL cells

**Figure 2:**
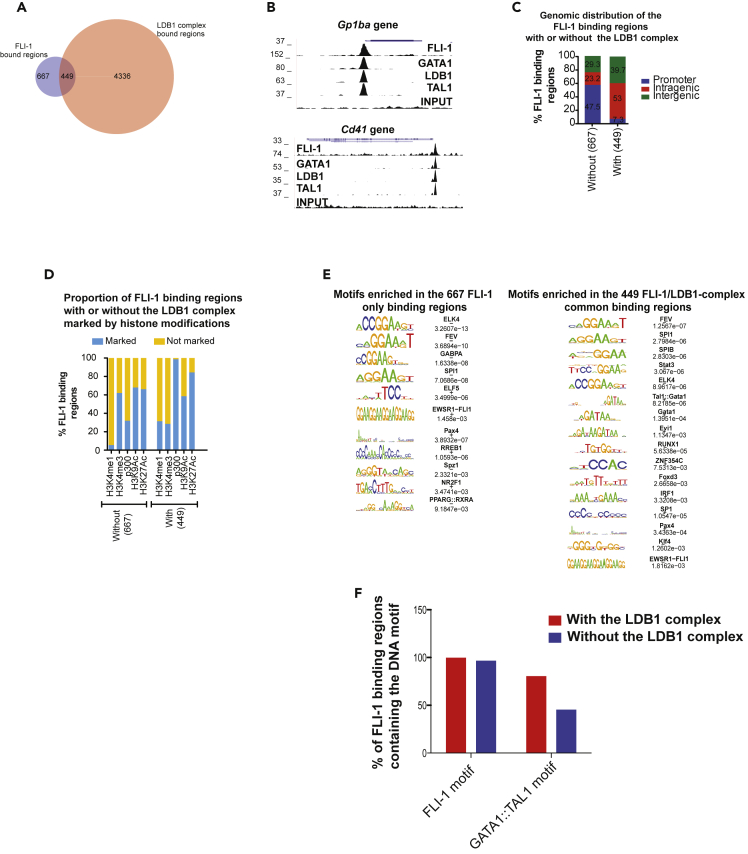
Fli-1 and the LDB1 complex mainly bind active enhancers in MEL cells (A) Venn diagram displaying the overlap between the binding regions of Fli-1 (blue circle) and the LDB1 complex (red circle) in MEL cells. (B) Genome view of the Fli-1, GATA1, LDB1, TAL1, and Input control ChIP-Seq data in MEL cells showing the co-recruitment of these proteins to the Gp1ba and Cd41 genes promoter. (C) Percentage of Fli-1 binding regions with or without the LDB1 complex located to promoters (blue), intragenic (red), or intergenic (green) regions in MEL cells. (D) Percentage of FLI-1 binding regions with or without the LDB1 complex marked (blue) or not marked (yellow) by H3K4me1, H3K4me3, P300, H3K9Ac, or H3K27Ac in MEL cells. (E) List of the motifs enriched in the Fli-1 only binding regions (left panel) or in the common binding regions (right panel). (F) Proportion of Fli-1 only (blue bars) or Fli-1/LDB1 complex commonly (red bars) bound regions containing the Fli-1 or the GATA1:TAL1 motif See also Figure S1 and Table S3.

Based on the abovementioned observations, we investigated whether Fli-1 and the LDB1 complex are recruited to common regions. We compared the Fli-1 genome-wide bound regions in MEL cells with those available for the LDB1 complex in the same cells (Soler et al., 2010). This analysis revealed that 449 regions recruit both Fli-1 and the LDB1 complex in MEL cells (Figures 2A and 2B). We confirmed by ChIP-qPCR the recruitment of the LDB1 complex on some FLI-1 binding regions (Figure S1D). Interestingly, although the Fli-1-bound regions without the LDB1 complex are mostly present at promoter regions, the 449 common binding regions are mostly present in intragenic and intergenic regions (Figure 2C). In agreement with this observation, the majority of the Fli-1-bound regions without the LDB1 complex are located at regions containing the H3K4me3, H3K9Ac, and H3K27Ac histone modifications corresponding to active promoters (Figure 2D). Although the majority of the commonly bound regions for Fli-1 and the LDB1 are marked by H3K9Ac and H3K27Ac histone signatures, they are almost always bound by the p300 acetyltransferase and are more often marked by H3K4me1 compared with the Fli-1 binding regions without the LDB1 complex (Figure 2D). These observations show that Fli-1 and the LDB1 complex mainly bind active enhancers.

Using comprehensive motif enrichment analyses, we identified that both Fli-1 only and common binding regions contain the ETS motif, as also identified above (Figures 2E and 2F). As expected, the common binding regions contain the TAL1:GATA1 motif when compared with the Fli-1-only binding regions (Figures 2E and 2F). This analysis also highlights the enrichment for other motifs such as RUNX1 or STAT3 motifs specifically found in the common binding regions, which suggests that these two TFs also contribute to the function of Fli-1 and the LDB1 complex.

Altogether, these data indicate that Fli-1 and the LDB1 complex mainly bind active enhancers in MEL cells, whereas Fli-1 without the LDB1 complex mainly bind active promoters.

### FLI-1 enables the recruitment of the LDB1 complex to enhancers and tethers the interaction to the targeted promoter

**Figure 3:**
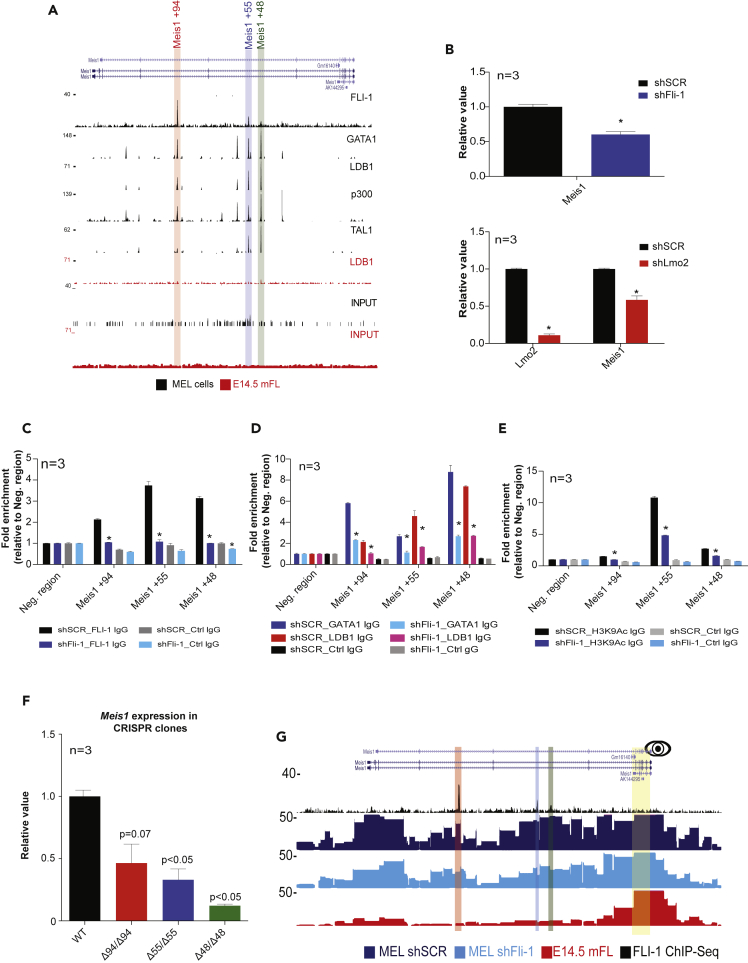
Fli-1 enables the recruitment of the LDB1 complex to 3 *Meis1* enhancers and promotes chromatin looping between these enhancers and the *Meis1* promoter in MEL cells (A) ChIP-Seq profiles of Fli-1, TAL1, GATA1, and LDB1 in MEL cells (black) and in fetal liver cells (red) showing three binding regions located 94, 55, and 48 kb downstream of the Meis1 promoter. (B) Top panel: Meis1 mRNA level from non-induced (dark color bars) and induced (light color bars) control (shSCR, black bars) or shFli-1 (blue bars) MEL cells. Bottom panel: Lmo2 and Meis1 mRNA level from control (shSCR, black bar) or shLmo2 transduced (red bar) MEL cells. The values are normalized to the value obtained for the Actb reference gene and those of control cells. Bars represent the geometric mean of three independent experiments. The error bars represent the standard error of the mean. ∗: p < 0.05 for the comparison between shSCR condition and the others (paired t test). (C–E) Fli-1 (C), GATA1, LDB1 (D), and H3K9Ac (E) ChIP-qPCR experiments from control (shSCR, black) or shFli-1 (blue) MEL cells and primers amplifying the control Amylase region (Amy), the Meis1 +94, +55, and +48 regions. The bars represent the geometric mean of three replicates. The error bars represent the standard error of the mean. ∗: p < 0.05 for the comparison between shSCR condition and the others (paired t test). (F) Meis1 mRNA levels from WT (black bar), Δ94/Δ94 (red bar), Δ55/Δ55 (blue bar), and Δ48/Δ48 (green bar) MEL cells. The values are normalized as in the panel c. The p values indicated above the bars have been obtained with a paired t test comparing WT MEL cells versus the Meis1 enhancer deleted CRISPR clones from three independent experiments. (G) Fli-1 ChIP-Seq (black) and T2C profile of control (dark blue) or shFli-1 (light blue) MEL cells and E14.5 fetal liver cells (red) in the Meis1 locus. The Meis1 promoter is used as a viewpoint indicated by the eye symbol. See also Figures S2 and S3.

The LDB1 complex is important for enhancer activity where it promotes chromatin looping with the associated promoter and the expression of the target genes (Soler et al., 2010; Stadhouders et al., 2012). However, except for the fusion protein EWS-FLI-1, the role of Fli-1 in these particular regions has not been addressed yet. To determine whether Fli-1 regulates the function of the LDB1 complex at enhancers regions, we focused on three regions in the *Meis1* locus bound by Fli-1 and the LDB1 complex, located at 94 (Meis1 +94), 55 (Meis1 +55), and 48 kb (Meis1 +48) downstream of the *Meis1* promoter (Figure 3A) and one region also bound by these proteins located 140 kb (Fut8-140) upstream of the *Fut8* promoter (Figure S2A). These genes were selected because of their known role in leukemia. In particular, *MEIS1* overexpression is very often observed in acute myeloid leukemia and is associated with poor prognosis (Argiropoulos et al., 2007; Honma et al., 2015; Kumar et al., 2009; Liu et al., 2017; Wang et al., 2014). Besides, Sasaki et al. showed that FUT8 represses erythroid differentiation of MEL cells, as FLI-1 (Sasaki et al., 2013). Given the role of Fli-1 in erythroleukemia cells, the regulation of these two genes could be part of the molecular mechanisms triggered by Fli-1 to transform the erythroid lineage. Although Fli-1 is co-localized in these regions in MEL cells with the LDB1 complex, p300, and histone marks suggesting that these are potential enhancers of these genes, in mouse fetal livers, which contain erythroid progenitors and precursors, Fli-1 and the LDB1 complex are not recruited to these particular regions (Figures S2B and S2C). As expected, the lack of the recruitment of these proteins is correlated with a lower expression of *Meis1* and *Fut8* in mouse fetal liver cells when compared with MEL cells (Figures S2D and S2E). These observations suggest that Fli-1 enables the recruitment of the LDB1 complex to these particular enhancers contributing to the activation of their expression in MEL cells.

To test this hypothesis, we first performed RT-qPCR experiments after repression of either *Fli-1* or *Lmo2* (whose depletion prevents the recruitment of the LDB1 complex to DNA (Inoue et al., 2013)) in MEL cells and checked for the expression of *Meis1* and *Fut8*. As observed in Figures 3B and S2F, the repression of *Fli-1* and *Lmo2* triggers the decrease of *Meis1* and *Fut8* mRNA levels, indicating that Fli-1 and the LDB1 complex activate the expression of these two genes in MEL cells. Strikingly, ChIP-qPCR experiments show that *Fli-1* repression in MEL cells significantly decreases the recruitment of both GATA1 and LDB1 to *Meis1 +94*, *Meis1+55*, *Meis1+48*, and *Fut8-140* regions, whereas GATA1 and LDB1 protein levels remain stable when compared with control cells (Figures 3C, 3D, S2G, and S2H). These experiments suggest that Fli-1 enables the recruitment or the stabilization of the LDB1 complex to these regions in MEL cells.

Similar ChIP-qPCR experiments show that in MEL cells, these regions are more often characterized by the deposition of the active histone mark H3K9Ac than in mouse fetal liver cells (Figure S2I) and that the repression of *Fli-1* decreases the level of this histone mark at all these four regions in MEL cells (Figures 3E and S2J), indicating that FLI-1 is important to maintain the active chromatin state. In addition to the recruitment of the p300 acetyltransferase, these observations suggest that these four regions act as enhancers of the *Meis1* and *Fut8* gene. To confirm this hypothesis, we used CRISPR/Cas9 system (Cong et al., 2013; Ran et al., 2013) to homozygously delete *Meis1+94*, *Meis+55*, or *Meis1+48* in MEL cells (Figure S3A) and checked for the expression of *Meis1* in these MEL cells clones. As a result, *Meis1* expression is decreased in these three clones compared with wild-type MEL cells, demonstrating that these three regions are indeed *Meis1* enhancers (Figure 3F).

Enhancers regions activate gene expression through chromatin looping with the target promoter (Kolovos et al., 2012). Because the LDB1 complex promotes looping of the DNA (Soler et al., 2010; Stadhouders et al., 2012), we investigated whether the decrease of *Meis1* expression observed after *Fli-1* repression is correlated with a change of the 3D conformation of this locus. Therefore, we performed a T2C analysis focusing on the *Meis1* locus in control MEL cells, *Fli-1* repressed MEL cells, and mouse fetal liver cells (Kolovos et al, 2014, 2018). The overall architecture of the *Meis1* locus remains generally unchanged in FLI-1 depleted cells compared with wild-type MEL cells (with the exception of some changes in the local interactome), whereas we observe major differences (such as fewer interactions) in mouse fetal liver cells (Figure S3B). These observations agree with the level of *Meis1* expression in mouse fetal liver cells and in Fli-1 depleted MEL cells, where *Meis1* is either completely or partially repressed, respectively (Figures 3B and S2D). When the *Meis1* promoter is taken as a viewpoint, we observed that the three enhancer regions bound by Fli-1 and the LDB1 complex are in close proximity with the *Meis1* promoter (Figures 3G and S3C). In contrast, in mouse fetal liver cells, we do not observe an interaction with the *Meis1* enhancers. This indicates that in mouse fetal liver cells, *Meis1* promoter and enhancers are not in close proximity. Finally, the close proximity between the *Meis1* promoter and enhancers is retained, albeit at a lesser level, upon *Fli-1* repression (Figures 3G and S3C). The same observations can be made when one of the three enhancers is used as viewpoints (Figure S3D). We also checked the local interactome of the *Fut8* locus by performing 3C-Seq experiments in the same conditions (Stadhouders et al., 2013). These experiments highlight that the *Fut8-140* and *Fut8* promoter regions are also in close proximity in MEL cells and that this proximity depends on Fli-1 (Figures S3E and S3F). In mouse fetal liver cells, these two regions are also in close proximity although at lower frequency compared with MEL cells, which correlate with *Fut8* expression in these two cell types.

Altogether, these data show that Fli-1 either enables the recruitment of the LDB1 complex or stabilizes this complex to enhancers commonly bound by these proteins. Moreover, Fli-1 is involved in their proximity with their target promoter through chromatin looping, and together, Fli-1 and the LDB1 complex activate the expression of these common target genes.

### Fli-1 and the LDB1 complex directly activate the expression of megakaryocyte genes in MEL cells and promote megakaryopoiesis

**Figure 4:**
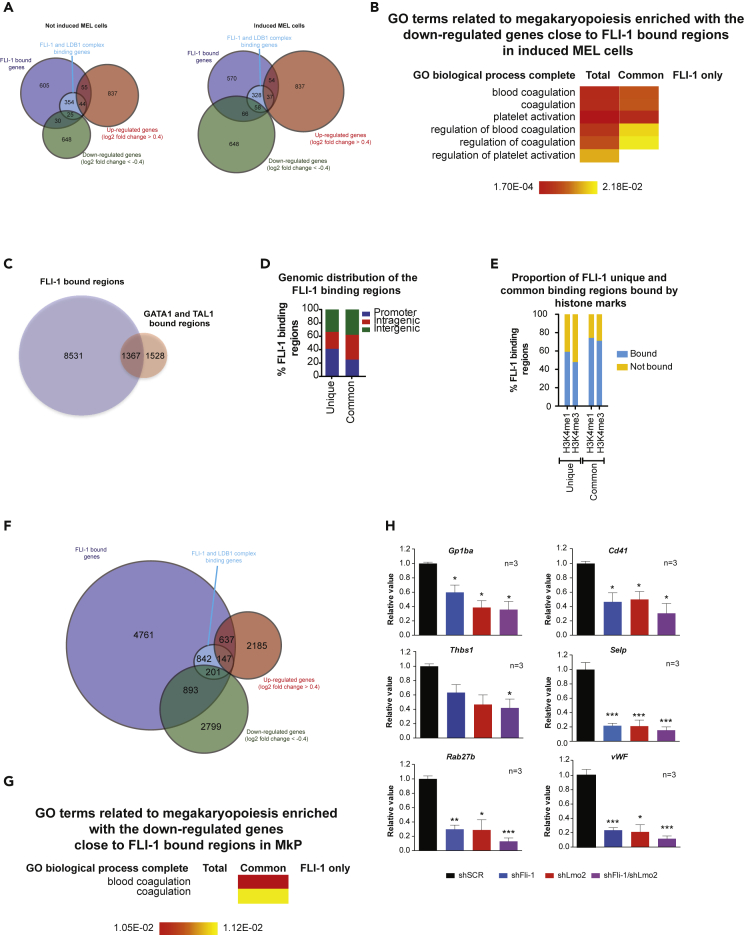
Fli-1 and the LDB1 complex activate megakaryocytic gene expression in MEL cells and megakaryocytes and promote megakaryopoiesis (A) Venn diagram displaying the overlap between the list of genes near the Fli-1 binding regions (dark blue circle) or near the Fli-1 and LDB1 complex common binding regions (light blue circle) and the list of downregulated genes (green circle) or upregulated genes (red circle) after *Fli-1* repression in non-induced (left panel) or in induced MEL cells (right panel). (B) GO terms relative to megakaryopoiesis enriched in the list of downregulated genes near FLI-1 binding regions in induced MEL cells (C) Venn diagram displaying the overlap between the binding regions of Fli-1 (blue circle) and the LDB1 complex (GATA-1 and TAL1 red circle) in megakaryocytes. (D) Percentage of Fli-1 binding regions with (common) or without (unique) the LDB1 complex located to promoters (blue), intragenic (red), or intergenic (green) regions in megakaryocytes. (E) Percentage of Fli-1 binding regions with (common) or without (unique) the LDB1 complex bound (blue) or not bound (yellow) by H3K4me1 or H3K4me3 in megakaryocytes. (F) Venn diagram showing the overlap between the genes close to Fli-1 binding regions (dark blue circle) or close to Fli-1 and LDB1 complex common binding regions (light blue circle) and the genes either downregulated (green circle) or upregulated (red circle) after Fli-1 repression in megakaryocytes. (G) GO terms relative to megakaryopoiesis enriched in the list of downregulated genes near FLI-1 binding regions in MkP. (H) Quantification of Gp1ba, Cd41, Thbs1, Selp, Rab27b, and vWF mRNA levels by RT-qPCR experiments from cells transduced with the shSCR (black bar), shFli-1 (blue bar), shLmo2 (red bar), or both (purple bar) lentiviruses. The bars represent the geometric mean of three independent experiments. The values are normalized to the value obtained in each condition for the Actb reference gene. The error bars represent the standard error of the mean. ∗: p < 0.05; ∗∗: p < 0.01; ∗∗∗: p < 0.005 for the comparison between shSCR condition and the others (paired t test). See also Figure S4 and Tables S4 and S5.

We finally addressed the influence of the Fli-1 and LDB1 complex binding on gene expression. We performed RNA-Seq experiments in non-induced and DMSO-induced MEL cells after *Fli-1* repression and we crossed the list of deregulated genes (log2 fold change >0.4 or < −0.4) with the list of genes targeted by either FLI-1 alone or commonly by the Fli-1/LDB1 complex. Overall, the overlap is small, suggesting that other proteins such as PU.1 are compensating the absence of FLI-1 (Juban et al., 2009) (Figure 4A). As already shown in previous studies, gene ontology analyses using PANTHER (Tables S5 and S6) show that Fli-1 directly activates the expression of genes involved in ribosome biogenesis independently of the recruitment of the LDB1 complex. Interestingly, several terms related to megakaryocyte function (blood coagulation etc) are enriched in the list of downregulated genes targeted by the Fli-1/LDB1 complex in induced MEL cells (Figure 4B), suggesting that Fli-1 and the LDB1 complex activate the expression of megakaryocytic genes in an erythroleukemic context. To check whether Fli-1 is important for the recruitment of the LDB1 complex to the regulatory sequences of these genes, as previously observed at enhancer regions, we performed GATA1 and LDB1 ChIP-qPCR experiments in non-induced and induced MEL cells after *Fli-1* repression at the *Gp1ba* and *Cd41* promoter regions, 2 known genes involved in megakaryopoiesis. This shows they are decreased when *Fli-1* is repressed, indicating that Fli-1 also enables the recruitment of the LDB1 complex or stabilizes it to regulatory sequences of megakaryocytic genes in MEL cells (Figure S4A).

These data suggest that Fli-1 and the LDB1 complex are important for megakaryopoiesis. To test this hypothesis, we analyzed the Fli-1, GATA1, and TAL1 genome-wide bound regions in megakaryocytes (Pimkin et al., 2014; Yue et al., 2014). We crossed the Fli-1 binding regions with the regions bound by both GATA1 and TAL1 (considered as the LDB1 complex). The number of Fli-1 binding regions in megakaryocytes is much higher than in MEL cells, suggesting that the chromatin is more permissive for Fli-1 in megakaryocyte. One thousand three hundred sixty seven regions are bound by these three proteins and therefore by Fli-1 and the LDB1 complex (Figure 4C). We confirmed by ChIP-qPCR the recruitment of Fli-1, GATA1, and LDB1 on some regions in the L8057 megakaryoblastic cell line (Figures S4B and S4C). As in MEL cells, Fli-1 binding regions are less present in promoters when it is with the LDB1 complex than without (Figure 4D). Moreover, Fli-1/LDB1 complex commonly bound regions are more frequently marked by active histone modifications (H3K4me1 and H3K4me3) than regions bound by Fli-1 alone, indicating that Fli-1 binds preferentially active regions when it is with the LDB1 complex (Figure 4E). We then performed RNA-Seq experiments in megakaryocyte progenitors (MkP) isolated from wild-type or *Fli-1* KO mice (Figure S4D) and crossed the list of misregulated genes with the list of FLI-1 bound regions with or without the LDB1 complex (Pronk et al., 2007; Starck et al., 2010). As in MEL cells, the overlap is relatively small, most likely because of compensatory mechanisms (Figure 4F). We performed gene ontology analyses using PANTHER (Table S7). Again, we identified terms related to megakaryopoiesis in the list of Fli-1/LDB1 complex commonly targeted genes, which are downregulated after *Fli-1* KO (Figure 4G), suggesting that Fli-1 and the LDB1 complex directly activate the expression of megakaryocytic genes and then contribute to megakaryopoiesis.

To test this hypothesis, we isolated cKit^+^ cells from mouse fetal liver cells, cultured them for 5 days in presence of mSCF and mTpo upon infection with lentiviruses expressing control shRNA or shRNA directed against *Fli-1* or *Lmo2* mRNA to repress their expression. After 4 more days of culture in presence of mTpo only, we isolated RNA and performed RT-qPCR experiments to quantify the expression of genes involved in terminal megakaryocyte differentiation and that are bound by Fli-1 and the LDB1 complex except for *Thbs1* (Figures S4E and S4F) (Chen et al., 2007). As expected, *Fli-1* and *Lmo2* expression are decreased when cells are infected with the lentiviruses expressing the shRNA directed against their mRNA (Figure S4G). *Fli-1* expression is decreased as well when *Lmo2* is repressed, suggesting that the LDB1 complex regulates *Fli-1* expression in megakaryocytes. Finally, the expression of the genes, which are activated upon terminal megakaryocyte differentiation, are also decreased after *Fli-1* and *Lmo2* repression (Figure 4H). Finally, to check whether Fli-1 is important for the recruitment of the LDB1 complex on the promoters of *Gp1ba* and *Cd41* megakaryocytic genes as it is in MEL cells, we performed ChIP-qPCR experiment in mouse fetal liver cells cultured with SCF and mTPO cytokines and infected with the lentiviruses expressing either control shRNA or shRNA against *Fli-1* mRNA. We confirmed the recruitment of Fli-1, GATA1, and LDB1 to the promoter of these two megakaryocytic genes in control cells (Figure S4H). In *Fli-1-*repressed fetal liver cells, we observed a decreased binding of Fli-1, GATA1, and LDB1 to these two promoters, suggesting that, as in MEL cells, Fli-1 either stabilizes or enables the recruitment of the LDB1 complex to megakaryocytic gene regulatory sequences (Figure S4H). Interestingly, these two particular regions are also bound by Fli-1, GATA1, and TAL1 in human megakaryocytes, suggesting that the mode of regulation of megakaryocytic genes by Fli-1 and the LDB1 complex is conserved in human.

Altogether, these data show that Fli-1 and the LDB1 complex directly co-activate the expression of megakaryocytic genes both in MEL cells and in megakaryocyte and suggest that Fli-1 and the LDB1 complex cooperate to promote megakaryopoiesis.

## Discussion

The aim of this study is to bring new insights about the molecular mechanisms involved in the several Fli-1 contributions to physiological and pathological development, especially in combination with the LDB1 complex. We show here that Fli-1 and the LDB1 complex mainly bind active enhancers in MEL cells and directly activate the expression of megakaryocytic genes promoting terminal megakaryocyte differentiation.

Fli-1 enables the recruitment of the LDB1 complex both at enhancers and regulatory sequences of megakaryocytic genes (Figures 3C–3E, S2G, and S4A). However, how Fli-1 contributes to maintain the LDB1 complex bound to DNA remains elusive. One explanation would be that Fli-1 acts as a pioneer TF for the LDB1 complex at these common binding regions. These particular TFs are characterized by their ability to bind chromatin in a repressed state and to open it by recruiting cofactors. The decrease of the H3K9Ac level in these common binding enhancer regions when FLI-1 is repressed is in favor with such a role of Fli-1 in MEL cells. A similar role has been described for Fli-1 in AML-ETO acute myeloid leukemia cells where the AML-ETO oncoprotein is recruited to pre-occupied Fli-1 bound regions (Martens et al., 2012). Nevertheless, we cannot exclude that Fli-1 actually stabilizes the LDB1 complex when bound to DNA. Interestingly, STAT3 DNA motif is only enriched in the regions bound by both Fli-1 and the LDB1 complex. GATA1 and phosphorylated STAT3 interact in MEL and human K562 cells and are both bound to gamma globin gene (Yao et al., 2009). However, the activation of the JAK-STAT signaling by IL-6, which increases the binding of STAT3 to these regions, is associated with a decrease of GATA1 binding and gamma globin gene silencing. These data show that phosphorylated STAT3 inhibits the recruitment of GATA1 at least at the gamma globin locus. Strikingly, the STAT3 DNA motif contains the ETS DNA binding motif recognized by Fli-1, which would suggest that Fli-1 and STAT3 are in competition to be recruited to this particular motif. Therefore, we hypothesize that FLI-1 prevents the recruitment of STAT3 at Fli-1/LDB1 complex binding regions hence favoring the recruitment of the LDB1 complex. Activating or repressing the JAK-STAT signaling pathway would then influence the interaction and the co-recruitment of Fli-1 and the LDB1 complex. Post-translational modifications of members of the LDB1 complex, especially of GATA1 have been shown to regulate their DNA-binding activity such as phosphorylation or acetylation (Lamonica et al., 2006; Partington and Patient, 1999). Another mechanism by which Fli-1 regulates the recruitment of the LDB1 complex to common binding regions would therefore be to recruit an enzyme that will modify the complex. Interestingly, the TRRAP acetyl-transferase appears in the top 10 of the Fli-1 protein partners identified by mass spectrometry (Giraud et al., 2014). TRRAP was already shown to interact with several TF and regulate their transcriptional activity (Liu et al., 2003). Whether TRRAP interacts with and acetylates GATA1 or another member of the LDB1 complex is still not known. Further experiments then have to be performed to decipher the potential contribution of TRRAP in the recruitment of the LDB1 complex.

We subsequently sought to investigate the role of Fli-1 in 3D chromatin conformation by studying two loci, *Meis1* and *Fut8* with three and one enhancer, respectively. We observed that the role of Fli-1 in 3D chromatin conformation, is loci dependent. The regulation of *Fut8* and promoter-enhancer interaction is dependent by the presence of FLI-1 (Figures S3A, S3F, and S3G). Notably, the promoter-enhancer interaction for *Meis1* appears to be stable and slightly affected by the binding of Fli-1 at its enhancers (Figures 3G and S3B–S3D). Therefore, we postulate that *Meis1* is stably in close proximity with its enhancers, potentially resembling a pre-looped conformation reported for other loci, where the promoter is continuously in close proximity with its enhancer, and only the presence of a TF (in this case Fli-1) activates the transcription of the gene (Kolovos et al., 2016). Therefore, most likely by enabling the recruitment of the LDB1 complex, which has a well-established role on those regions, Fli-1 promotes chromatin looping between the enhancers bound by both Fli-1 and the LDB1 complex and their target promoter. *Meis1* and *Fut8* gene, which are activated by Fli-1 and the LDB1 complex bound at their enhancer regions, encode two proteins, which have functions in different cancer. Indeed, *Meis1* aberrant expression actively contributes to acute myeloid leukemia, whereas *Fut8* overexpression has been found in non-hematopoietic cancer (Argiropoulos et al., 2007; Honma et al., 2015; Kumar et al., 2009; Liu et al., 2017; Wang et al., 2014). Besides, FUT8 inhibits erythroid differentiation of MEL cells and K562 cells, which suggests that FUT8 also contributes to erythroleukemia (Sasaki et al., 2013). Therefore, targeting enhancers of genes involved in leukemia would be one mechanism by which Fli-1 contributes to erythroleukemia. In addition to bringing new insights concerning Fli-1 contributions to cancer, our data identify three new enhancers of *Meis1* in erythroleukemia. As mentioned earlier, *Meis1* expression promotes leukemia development. Nevertheless, it is still unclear how *Meis1* is overexpressed in such pathological condition. Testing whether Fli-1 and the LDB1 complex co-regulate *MEIS1* in this context would be interesting for therapeutic purpose.

Finally, we showed that Fli-1 and the LDB1 complex directly activate the expression of megakaryocytic genes both in MEL cells and in megakaryocytes and promote terminal megakaryocyte differentiation (Figures 4 and S5). The activation of genes involved in megakaryopoiesis in an erythroleukemic context supposes that MEL cells have an increased plasticity. This increased plasticity has been found as well in mice overexpressing another ETS TF, ERG, whose ETS DNA-binding domain has 98% homology with the one of Fli-1. These mice develop among other type of leukemia, acute erythroleukemia. When plated in methylcellulose with the appropriate cytokines, these erythroleukemic cells give rise to megakaryocytic colonies, showing that these cells kept the ability to express the megakaryocytic program (Carmichael et al., 2012). Whether keeping the megakaryocytic potentiality is a common feature in human acute erythroleukemia (AML-M6) has never been addressed yet. But we think that first checking whether AML-M6 cells still express megakaryocytic genes and developing strategies to repress it would improve the current therapies against such leukemia. Next to being co-recruited in terminally differentiated cells, Fli-1 and the LDB1 complex share already common binding regions in immature progenitor cells such as hemogenic endothelium and multipotent hematopoietic progenitors, suggesting that they also interplay at early stages of hematopoiesis. Recently, hematopoietic stem cells with a megakaryocytic bias have been identified (Shin et al., 2014). Whether Fli-1 and the LDB1 complex play a role in these HSC have not been addressed yet, but seeing our data and the aforementioned data, we suspect that these proteins are involved in priming those cells toward megakaryocytes. As already mentioned, a cocktail composed of Fli-1, GATA1, and TAL1 have been used to enhance megakaryocyte production (Moreau et al., 2016). We propose to improve this strategy by adding both LDB1 and LMO2.

### Limitations of the study

The results in this study are obtained in murine transformed cell lines or mouse primary cells. Although the FLI-1 and the LDB1 complex binding sites shown in this study seem to be conserved in humans, the data presented in this work might not entirely reflect what happens in humans. Therefore, tackling the interplay between FLI-1 and the LDB1 complex in human primary cells will definitely validate its role in leukemia and megakaryopoiesis.

### Resource availability

#### Lead contact

Dr Guillaume GIRAUD—guillaume.giraud@inserm.fr.

#### Materials availability

The study did not generate new unique reagents.

#### Data and code availability

The accession number for the FLI-1 ChIP-Seq, the RNA-Seq and the T2C experiments reported in this paper is SRA: SRP158024.

## Methods

All methods can be found in the accompanying transparent methods supplemental file.

## Supplemental information

Document S1. Transparent methods, Figures S1–S4, and Tables S1 and S2

Table S3. Coordinates of the FLI-1 binding regions in MEL cells and megakaryocytes, related to Figures 1 and 2

Table S4. List of misregulated genes after Fli-1 repression in MEL cells and MkP, related to Figure 4

Table S5. GO terms enriched in the list of upregulated genes near FLI-1 binding regions in non-induced MEL cells, related to Figure 4

Table S6. GO terms enriched in the list of misregulated genes in induced MEL cells near FLI-1 binding regions, related to Figure 4

Table S7. GO terms enriched in the list of misregulated genes near FLI-1 binding regions in MkP, related to Figure 4
