# Supplementary material for: Interplay between FLI-1 and the LDB1 complex in murine erythroleukemia cells and during megakaryopoiesis
Source: iScience. 2021 Feb 20;24(3):102210. doi: 10.1016/j.isci.2021.102210 (PMC7940982; doi:10.1016/j.isci.2021.102210)
Supplement: Document S1. Transparent methods, Figures S1–S4, and Tables S1 and S2 [file mmc1.pdf]

iScience, Volume *24*

Supplemental information

Interplay between FLI-1 and the LDB1 complex in murine erythroleukemia cells and during megakaryopoiesis

Guillaume Giraud, Petros Kolovos, Ilias Boltsis, Jente van Staalduinen, Boris Guyot, Michele Weiss-Gayet, Wilfred van IJcken, François Morlé, and Frank Grosveld

SUPPLEMENTAL FIGURE LEGEND

Figure S1 (Related to Figure 1 and Figure 2) : FLI-1 and the LDB1 complex are co-recruited in MEL cells

a ChIP experiments performed with either the anti-FLI-1 antibody or the control IgG in non induced or induced MEL cells expressing either control shRNA (shSCR) or shRNA against the Fli-1 mRNA (shFli-1) followed by qPCR to amplify the control region in the β-amylase locus (Amy, black bar) or the FLI-1 binding regions identified by ChIP-Seq (see Figure 1d) in the Gp1ba (blue bar) and the Cd41 promoter (red bar) and in the Tgfb1 (green bar), Stat1 (orange bar), Trp53 (purple bar) and Pdcd1lg2 yellow bar) loci. The bars represent the geometric mean of the ratio between the FLI-1 binding regions and the control region of 3 independent experiments. The error bars represent the standard errors. *: $p<0.05$ for the comparison between shSCR and shFli-1 conditions (paired t-test).

b Western blot analyses of the FLI-1 (bottom blot) or the VCP (top blot, loading control) protein levels in non-induced (lanes 1 and 2) and induced (lanes 3 and 4) MEL cells expressing the control shRNA (shSCR, lanes 1 and 3) or the shRNA against Fli-1 mRNA (shFli-1, lanes 2 and 4). The blots are representative of 3 independent experiments.

c RT-qPCR analyses of the Fli-1 mRNA levels in non-induced and induced MEL cells expressing the control shRNA (shSCR, black bars) or the shRNA against Fli-1 mRNA (shFli-1, blue bars). The bars represent the geometric mean of the ratio of the signals between shFli-1 and shSCR normalized to the signals of the Actb reference gene of 3 independent experiments. The error bars represent the standard errors. *: $p<0.05$ for the comparison between shSCR and shFli-1 conditions (paired t-test).

d ChIP experiments performed with either the anti-GATA1 or the anti-LDB1 antibodies or the control IgG in non induced or induced MEL cells followed by qPCR to amplify the control region in the β-amylase locus (Amy, black bar) or the FLI-1 binding regions identified by ChIP-Seq (see Figure 1d) in the Gp1ba (blue bar) and the Cd41 promoter (red bar). The bars represent the geometric mean of the ratio between the FLI-1 binding regions and the control region of 3 independent experiments. The error bars represent the standard error.

Figure S2 (Related to Figure 3) : FLI-1 and the LDB1 complex directly activate *Fut8* and *Meis1* expression by binding to enhancers in MEL cells

a ChIP-Seq profiles of FLI-1, GATA1, LDB1, TAL1, LMO2, p300 and the Input control in MEL cells (black) and LDB1 and Input control in E14.5 mFL (red) on the Fut8 locus.

b ChIP experiments with the anti-FLI-1 (top panel), anti-GATA1 and anti-LDB1 (bottom panel) antibodies and their respective control IgG in MEL cells and E14.5 fetal liver cells (mFL) followed by qPCR experiments to amplify the amylase control region (Amy, black bar), the Meis1 +94 (red bar), the Meis1 +55 (blue bars) or the Meis1 +48 (green bars) regions. The bars represent the geometric mean of the ratio between the FLI-1 binding regions and the control region of 3 independent experiments. The error bars represent the standard error. *: $p<0.05$ for the comparison between MEL cells and fetal liver cells.

c ChIP-qPCR experiments performed with either an anti-FLI-1 (top panel), an anti-GATA1 or an anti-LDB1 (bottom panel) antibody and their respective control IgG in MEL cells and E14.5 mFL followed by qPCR amplifying the control Amylase region (black bars) and the Fut8-140 region (blue bar). The bars represent the geometric mean of the ratio between the FLI-1 binding regions and the control region of 3 independent experiments. The error bars represent the standard error. *: $p<0.05$ for the comparison between MEL cells and fetal liver cells.

d Quantification of the Meis1 mRNA levels by RT-qPCR experiments in E14.5 fetal liver (red bar) and MEL (blue bar) cells. The bars represent the geometric mean of the ratio between MEL cells and fetal liver cells. The error bars represent the standard error. *: $p<0.05$ for the comparison between MEL cells and fetal liver cells.

e Quantification of the Fut8 mRNA levels by RT-qPCR experiments in E14.5 fetal liver (red bar) and MEL (blue bar) cells. The bars represent the geometric mean of the ratio between MEL cells and fetal liver cells. The error bars represent the standard errors. *: $p<0.05$ for the comparison between MEL cells and fetal liver cells.

f Left panel: Fut8 mRNA level from non-induced control (shSCR, black bars) or shFli-1 (blue bars) MEL cells. Right panel: Lmo2 and Meis1 mRNA level from control (shSCR, black bar) or shLmo2 transduced (green bar) MEL cells. The values are normalized to the value obtained for the Actb reference gene and those of control cells. Bars represent the geometric mean of 3 independent experiments. The error bars represent the standard errors. *: $p<0.05$ for the comparison between shSCR condition and the others (paired t-test).

g ChIP-qPCR experiments using an anti-FLI-1 (top panel), an anti-LDB1 (middle panel) or an anti-

GATA1 (bottom panel) antibody and their respective control IgG in non –induced and induced control (shSCR) or shFli-1 MEL cells followed by qPCR amplifying the control Amylase region (black bars) and the Fut8-140 region (blue bar). The bars represent the average of the ratio between the FLI-1 binding regions and the control region of 3 independent experiments. The error bars represent the standard error. *: p<0.05 for the comparison between shSCR and shFli-1 conditions (paired t-test).

h Western blot analyses of GATA1 (2nd top panel), LDB1 (bottom panel) VCP (loading control, top panel and 2nd bottom panel) protein levels in non-induced (lanes 1 and 2) and induced (lanes 3 and 4) MEL cells expressing the control shSCR (lanes 1 and 3) or shFli-1 (lanes 2 and 4). The pictures are representative of 3 independent experiments.

i Left panel : ChIP experiment using an anti-H3K9Ac antibody or its control IgG from MEL cells or E14.5 mouse fetal liver cells followed by qPCR experiments to amplify the *Meis1+94* (red bars), the *Meis1+55* (blue bars) or the *Meis1+48* (green bars) regions. Right panel : ChIP experiment using an anti-H3K9Ac antibody or its control IgG from MEL cells or E14.5 mouse fetal liver cells followed by qPCR experiments to amplify the negative region (Amy, black bars) or the *Fut8-140* (blue bars) region. In the two panels, the bars represent the average of the relative value compared to Input of 3 independent experiments. The error bars represent the standard error. *: p<0.05 for the comparison between MEL cells and fetal liver cells.

j ChIP-qPCR experiments performed with an anti-H3K9Ac or its respective control IgG in control (shSCR) or shFli-1 non-induced or induced MEL cells followed by qPCR amplifying the control Amylase region (black bars) and the Fut8-140 region (blue bar). The bars represent the average of the relative value compared to Input of 3 independent experiments. The error bars represent the standard error. *: p<0.05 for the comparison between control and shFli-1 cells.

Figure S3 (Related to Figure 3) : FLI-1 and the LDB1 complex reshape the 3D chromatin landscape of *Meis1* and *Fut8* loci.

a Left panel: Scheme of the Meis1 locus. The promoter and the sense of transcription is indicated by an arrow. The Meis1 +48, Meis1 +55 and the Meis1 +94 regions are indicated by a green, blue and red rectangle respectively. The gRNA used to delete these 3 regions individually are indicated by scissors and the primers couple used to screen for clones having the deletion of the Meis1 +48, Meis1 +55 and the Meis1 +94 regions are indicated by

green, blue and red lanes respectively. Right panel: PCR using the primer couples mentioned above from gDNA of WT, Δ94/Δ94, Δ55/Δ55 and Δ48/Δ48 clones.

b T2C profiles on the Meis1 locus from MEL cells expressing either control shRNA (shSCR, middle panel) or shRNA targeting Fli-1 mRNA (shFli-1, top panel) or from mouse fetal liver cells (E14.5 mFL, bottom panel). Below are plotted the LDB1, GATA1, H3K27Ac, CTCF ChIP-Seq profiles and the DNaseI profile. The picture is representative of 2 technical duplicates.

c FLI-1 ChIP-Seq and differential contact profiles between Control and shFli-1 MEL cells (blue line) or between MEL cells and E14.5 mFL (orange line). The lines represent the log2 fold change of the aforementioned differences. The *Meis1* promoter is taken as a viewpoint.

d FLI-1 ChIP-Seq (black) and T2C plotted as 3C-Seq profile profile view of MEL cells expressing the control shRNA (dark colors) or shRNA against Fli-1 mRNA (medium colors) and E14.5 fetal liver cells (light colors) in the Fut8 locus. The Meis1 +94 enhancer (red profiles), Meis1 +55 (blue profiles) and Meis1 +48 (green profiles) are used as a viewpoint.

e FLI-1 ChIP-Seq (black) and 3C-Seq profile view of MEL cells expressing the control shRNA (dark blue) or shRNA against Fli-1 mRNA (light blue) and E14.5 fetal liver cells (red) in the Fut8 locus. The Fut8 promoter is used as a viewpoint.

f FLI-1 ChIP-Seq (black) and 3C-Seq profile view of MEL cells expressing the control shRNA (dark blue) or shRNA against Fli-1 mRNA (light blue) and E14.5 fetal liver cells (red) in the Fut8 locus. The Fut8 -140 region is used as a viewpoint.

Figure S4 (Related to Figure 4) : FLi-1 and the LDB1 complex directly activate the expression of megakaryocytic genes

a ChIP experiments using an anti-GATA-1 or an anti-LDB1 antibody or their respective control IgG performed in non-induced and induced MEL cells expressing a control shRNA (shSCR) or shRNA directed against Fli-1 mRNA (shFli-1) followed by qPCR to amplify the control region in the β-amylase locus (Amy, black bar) or the FLI-1 binding regions identified by ChIP-Seq (see Figure 1d) in the Gp1ba (blue bar) and the Cd41 promoter (red bar). The bars represent the geometric mean of the ratio between the FLI-1 binding regions and the control region of 3 independent experiments. The error bars represent the standard error. *: p<0.05 for the comparison between shSCR and shFli-1 MEL cells (paired t-test).

b, c (b) ChIP experiments performed with either the anti-FLI-1 antibody (blue bar) or the control IgG (black bar) in L8057 cells followed by qPCR to amplify the control region in the β -amylase locus (Amy) or the FLI-1 binding regions identified by ChIP-Seq (see Figure 1d) in the Gp1ba, Cd41 loci. The bars represent the geometric mean of the ratio between the FLI-1 binding regions and the control region of 3 independent experiments. The error bars represent the standard error.*: $p<0.05$ for the comparison between FLI-1 and control IgG (paired t-test). (c): ChIP experiments with antibodies against GATA-1 (red bars) or LDB1 (orange bars) or the control IgG (black bars) in L8057 cells followed by qPCR to amplify the control amylase region (Negative region), the Gp1ba promoter and the Cd41 promoter. The bars represent the geometric mean of the ratio between the FLI-1 binding regions and the control region (of 3 independent experiments. The error bars represent the standard error. * : p-value <0.05 for the comparison between FLI-1 binding regions and the negative region (paired t-test).

d Top panel: Genome view of the RNA-Seq reads of WT (bottom) or the FLI-1 KO MkP (top) displaying the absence of reads in the coding sequence of the Fli-1 exon 9 in the FLI-1 KO MkP. Bottom panel: Fli-1 RPKM value in WT (black bar) and FLI-1 KO (purple bar) MkP.

e Scheme of the protocol followed to analyze gene expression in megakaryocytes after Fli-1 and/or Lmo2 repression. cKit+ cells were isolated from E14.5 mouse fetal liver and infected with lentiviruses expressing either a control shRNA (shSCR) or shRNA targeting Fli-1 or Lmo2 or both mRNA. Cells were then cultured for 5 days in presence of mSCF and mTpo followed by 4 extra days in presence of only mTpo. RNA were isolated and RT-qPCR were performed to assess the mRNA levels of megakaryocytic genes (Figure 4f).

f Quantification by RT-qPCR analyses of the Fli-1 (left panel) and Lmo2 (right panel) mRNA levels from control mature megakaryocytes (black bars) or mature megakaryocytes knocked-down for Fli-1 (shFli-1, blue bars), or for Lmo2 (shLmo2, red bars) or both (purple bars). The bars represent the geometric mean of 3 independent experiments. The error bars represent the standard errors. **: $p<0.01$ for the comparison with the shSCR condition (paired t-test).

g ChIP-Seq profiles for FLI-1, GATA1 and TAL1 and the Input control at the Gp1ba, Cd41, Selp, Rab27b and vWF loci in megakaryocytes.

h Fetal liver cells from E14.5 mouse embryos have been infected by lentiviruses expressing either control shRNA (shSCR, dark coloured bars) or shRNA against *Fli-1* mRNA (light coloured bars) and cultured for 3 days in presence of SCF and Tpo cytokines to prime megakaryopoiesis. Cells were then crosslinked and ChIP experiments were performed using either the anti-FLI-1 (blue bars), the anti-GATA1 (red bars) and the anti-LDB1 (orange bars) antibodies followed by qPCR experiments to amplify the negative region (in the β-amylase locus) and the promoters of *Gp1ba* and *Cd41* gene. The bars represent the fold enrichment relative to the control IgG that have been used in parallel.

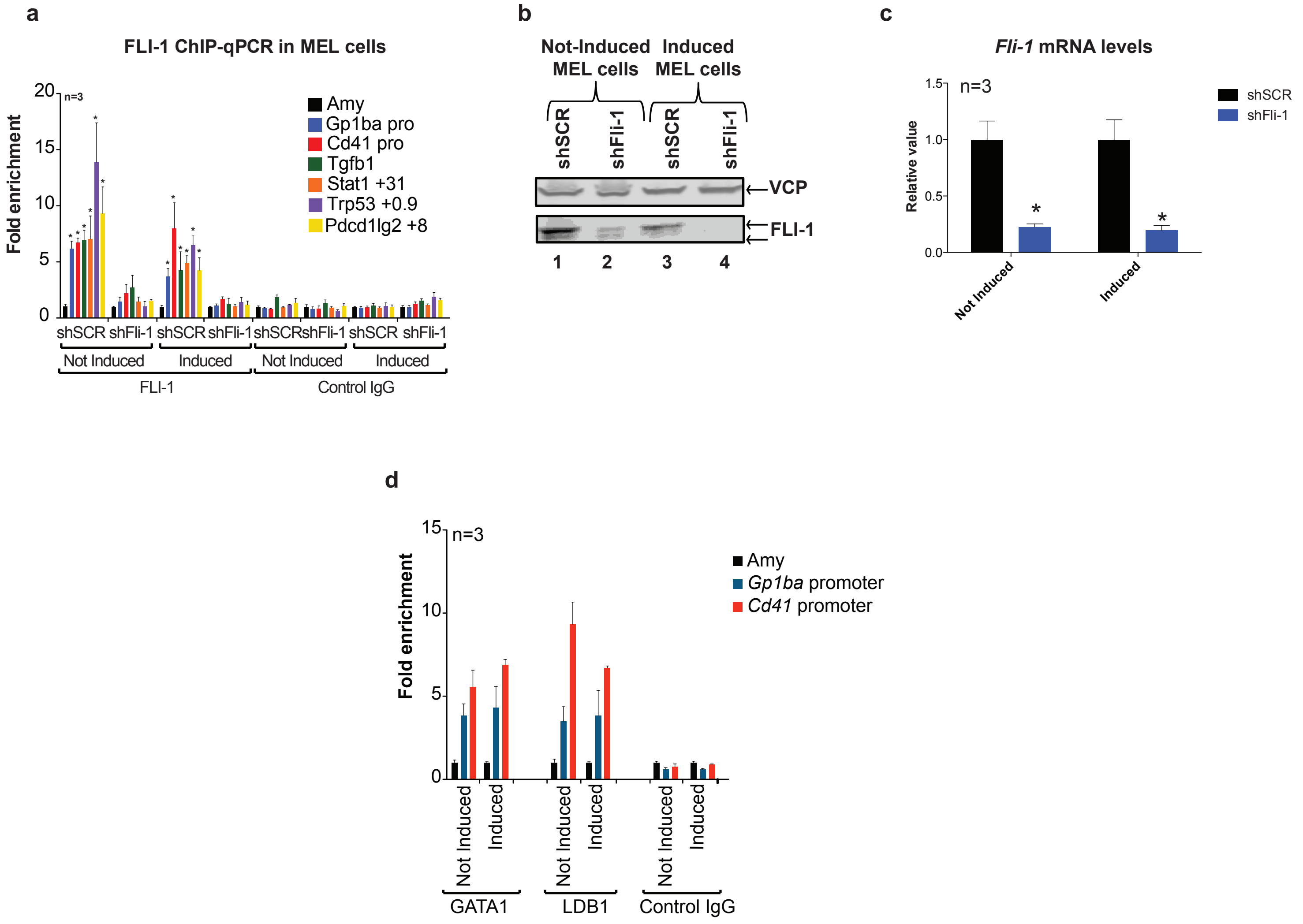
a
FLI-1 ChIP-qPCR in MEL cells
n=3
Fold enrichment
Amy
Gp1ba pro
Cd41 pro
Tgfb1
Stat1 +31
Trp53 +0.9
Pdcd1lg2 +8
shSCR shFli-1 shSCR shFli-1 shSCR shFli-1 shSCR shFli-1
Not Induced Induced Not Induced Induced
FLI-1
Control IgG
b
Not-Induced MEL cells
Induced MEL cells
shSCR
shFli-1
shSCR
shFli-1
VCP
FLI-1
1 2 3 4
c
Fli-1 mRNA levels
n=3
Relative value
shSCR
shFli-1
Not Induced
Induced
d
n=3
Fold enrichment
Amy
Gp1ba promoter
Cd41 promoter
Not Induced
Induced
GATA1
LDB1
Control IgG

Figure S1

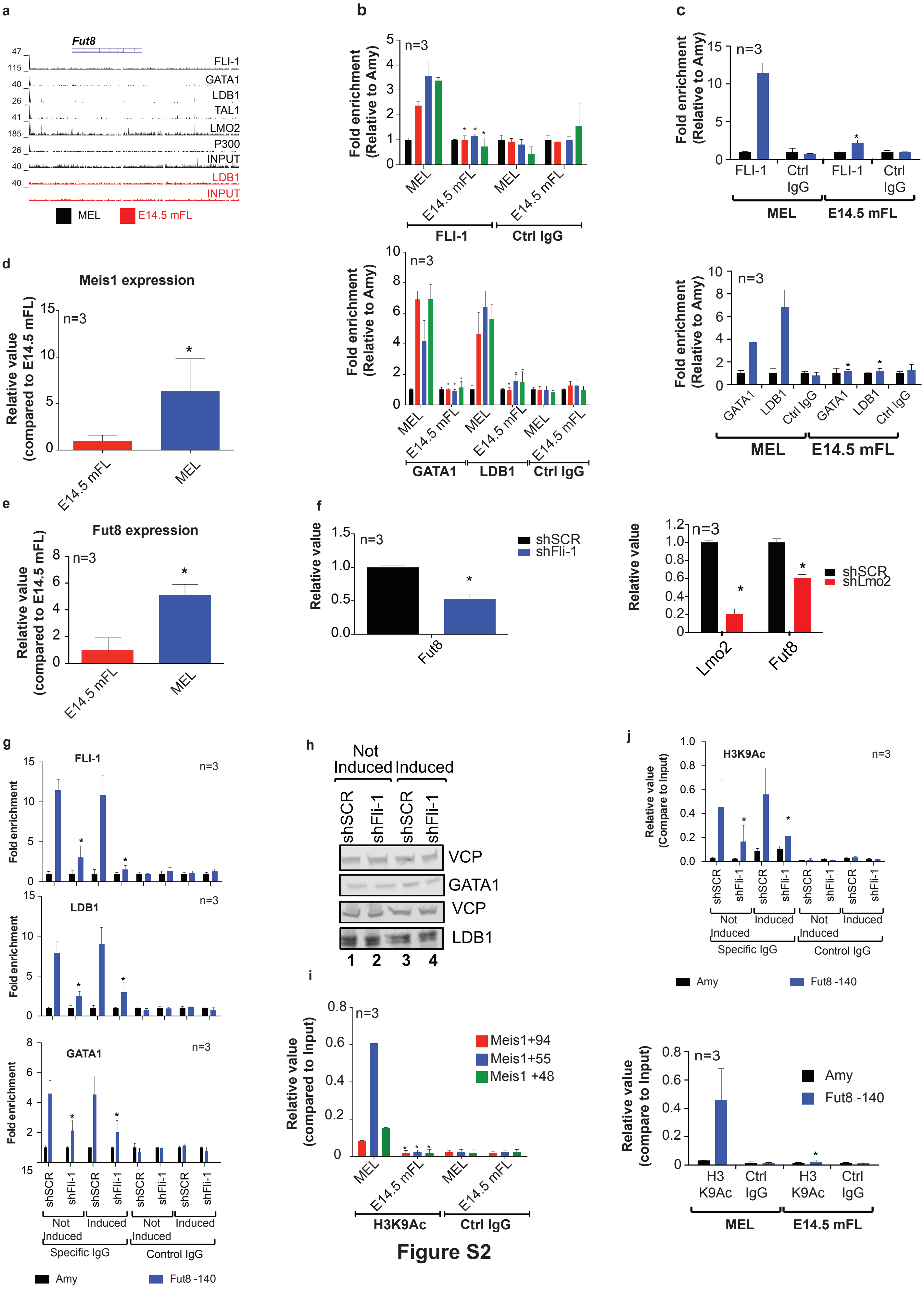
a
Fut8
FLI-1
GATA1
LDB1
TAL1
LMO2
P300
INPUT
LDB1
INPUT
MEL
E14.5 mFL
b
Fold enrichment (Relative to Amy)
n=3
MEL
E14.5 mFL
FLI-1
Ctrl IgG
GATA1
LDB1
c
FLI-1
Ctrl IgG
MEL
E14.5 mFL
d
Meis1 expression
Relative value (compared to E14.5 mFL)
e
Fut8 expression
f
Relative value
shSCR
shFli-1
Fut8
shLmo2
Lmo2
g
FLI-1
Fold enrichment
shSCR
shFli-1
Not Induced
Induced
Specific IgG
Control IgG
Amy
Fut8 -140
h
VCP
GATA1
LDB1
1 2 3 4
i
Relative value (compared to Input)
Meis1+94
Meis1+55
Meis1 +48
H3K9Ac
j
Relative value (Compare to Input)
H3 K9Ac
Ctrl IgG

Figure S2

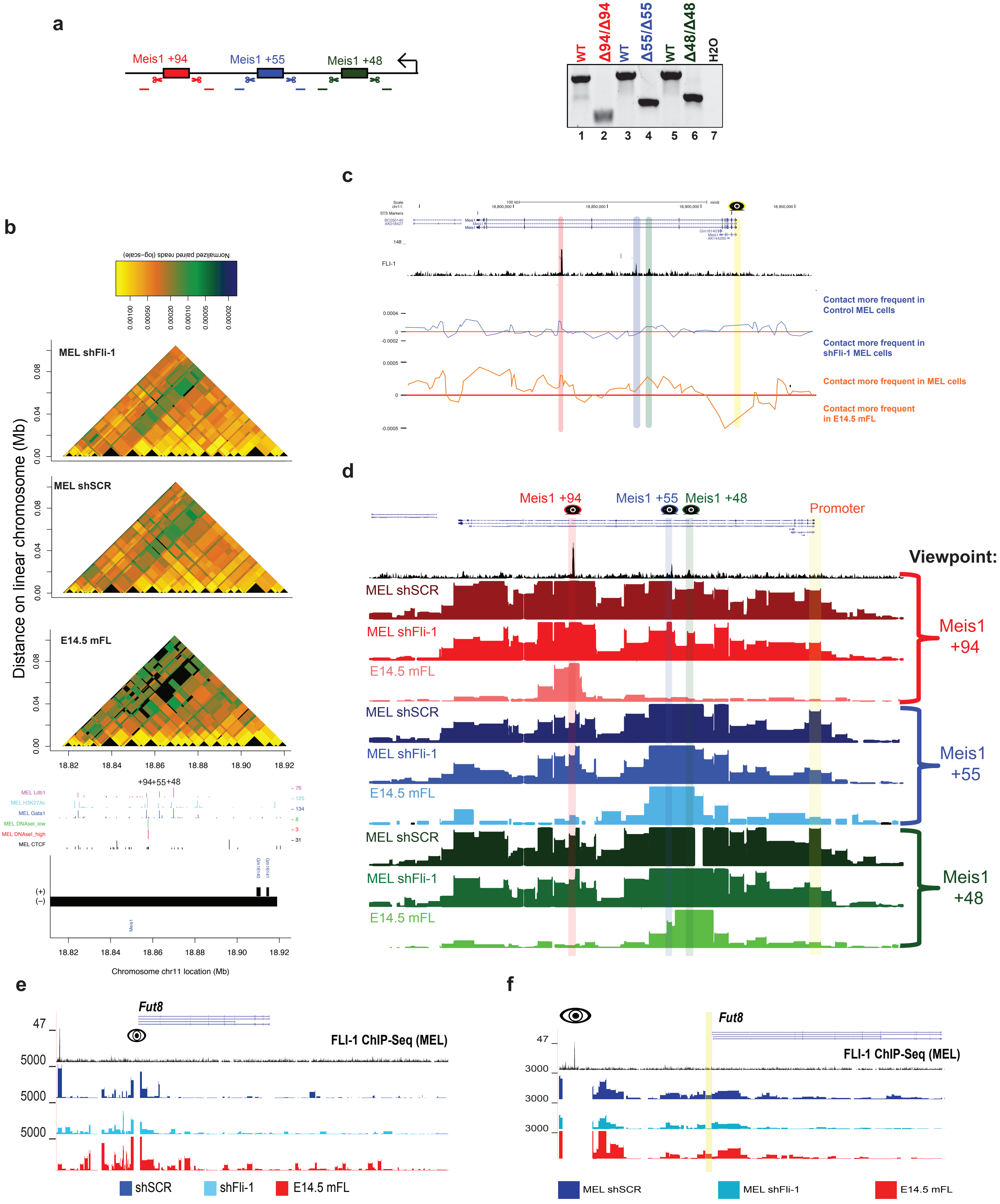
a
Meis1 +94
Meis1 +55
Meis1 +48
WT
Δ94/Δ94
WT
Δ55/Δ55
WT
Δ48/Δ48
H2O
1 2 3 4 5 6 7
b
Normalized paired reads (log-scale)
0.00100
0.00050
0.00020
0.00010
0.00005
0.00002
MEL shFli-1
MEL shSCR
E14.5 mFL
Distance on linear chromosome (Mb)
18.82
18.84
18.86
18.88
18.90
18.92
+94+55+48
MEL Ldb1
MEL H3K27Ac
MEL Gata1
MEL DNaseI_low
MEL DNaseI_high
MEL CTCF
Chromosome chr11 location (Mb)
c
FLI-1
Contact more frequent in Control MEL cells
Contact more frequent in shFli-1 MEL cells
Contact more frequent in MEL cells
Contact more frequent in E14.5 mFL
d
Meis1 +94
Meis1 +55
Meis1 +48
Promoter
Viewpoint:
MEL shSCR
MEL shFli-1
E14.5 mFL
Meis1 +94
Meis1 +55
Meis1 +48
e
Fut8
FLI-1 ChIP-Seq (MEL)
47
5000
shSCR
shFli-1
E14.5 mFL
f
Fut8
FLI-1 ChIP-Seq (MEL)
47
3000
MEL shSCR
MEL shFli-1
E14.5 mFL

Figure S3

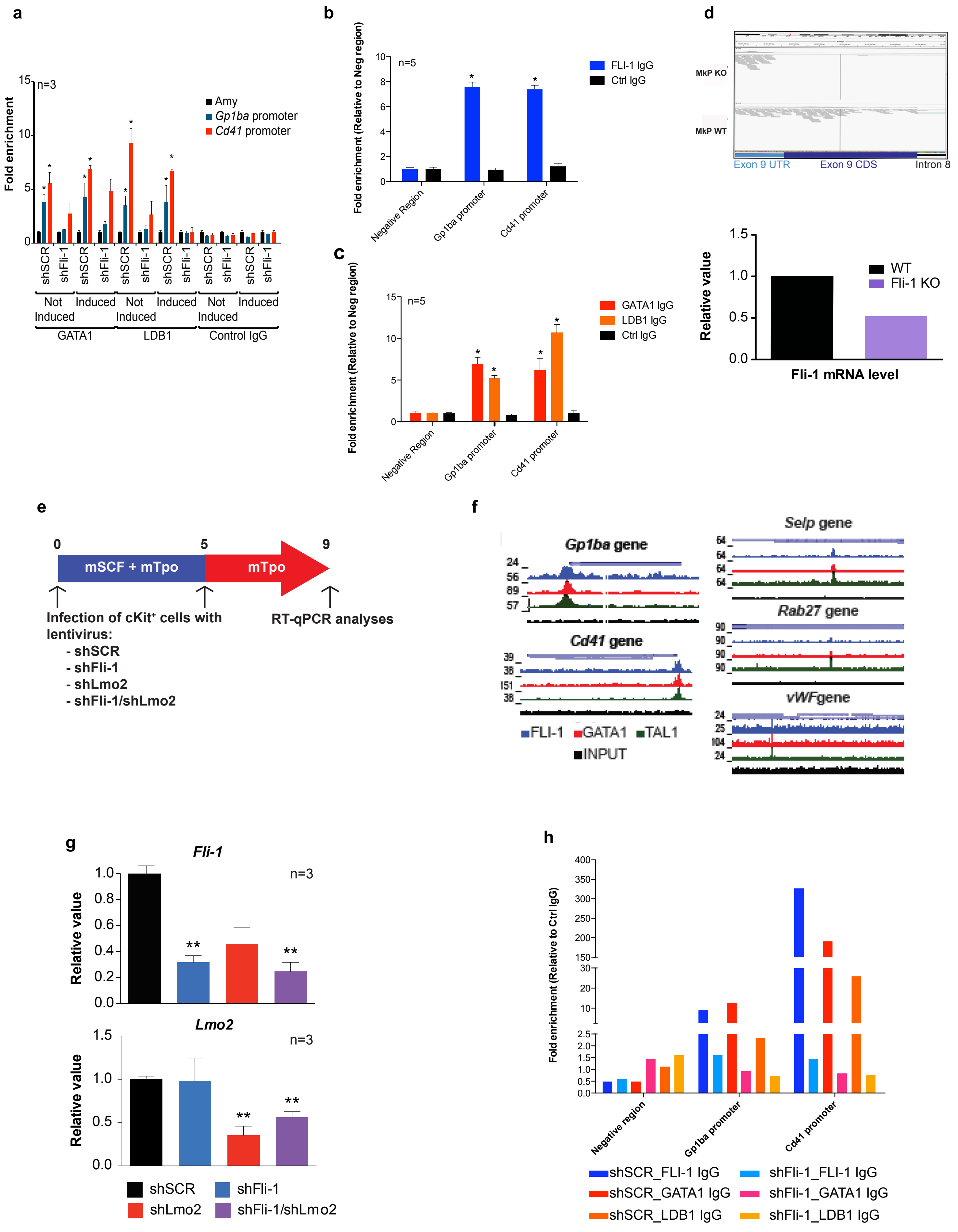

a
n=3
Fold enrichment
Amy
Gp1ba promoter
Cd41 promoter
shSCR
shFli-1
Not Induced
Induced
GATA1
LDB1
Control IgG
b
n=5
Fold enrichment (Relative to Neg region)
FLI-1 IgG
Ctrl IgG
Negative Region
Gp1ba promoter
Cd41 promoter
c
n=5
GATA1 IgG
LDB1 IgG
Ctrl IgG
d
MkP KO
MkP WT
Exon 9 UTR
Exon 9 CDS
Intron 8
Relative value
WT
Fli-1 KO
Fli-1 mRNA level
e
0
5
9
mSCF + mTpo
mTpo
Infection of cKit+ cells with lentivirus:
- shSCR
- shFli-1
- shLmo2
- shFli-1/shLmo2
RT-qPCR analyses
f
Gp1ba gene
Cd41 gene
Selp gene
Rab27 gene
vWFgene
FLI-1
GATA1
TAL1
INPUT
g
Fli-1
Lmo2
n=3
Relative value
shSCR
shFli-1
shLmo2
shFli-1/shLmo2
h
Fold enrichment (Relative to Ctrl IgG)
Negative region
Gp1ba promoter
Cd41 promoter
shSCR_FLI-1 IgG
shSCR_GATA1 IgG
shSCR_LDB1 IgG
shFli-1_FLI-1 IgG
shFli-1_GATA1 IgG
shFli-1_LDB1 IgG

Figure S4

SUPPLEMENTAL TABLE

Table S1 : List of antibodies used (Related to Figure 1, 2 3 and 4)

Antibody against	Techniques	Conjugated with	Brand	Reference
CD117	FACS	PE-Cy7	BioLegend	105813
		APC	BioLegend	105812
CD41	FACS	FITC	BioLegend	133903
CD61	FACS	PE	BioLegend	104307
TER119	FACS	Biotin	BioLegend	116203
SCA1	FACS	Biotin	BioLegend	108103
CD3	FACS	Biotin	BioLegend	100243
CD127	FACS	Biotin	BioLegend	135005
CD19	FACS	Biotin	BioLegend	101504
Streptavidin	FACS	APC	BioLegend	405207
CD150	FACS	PerCp-Cy5.5	BioLegend	115921
FLI-1	Western blot/ChIP	NA	Abcam	ab-15289
GATA1	Western blot/ChIP	NA	Santa Cruz Biotechnologies	sc-1234
LDB1	Western blot/ChIP	NA	Santa Cruz Biotechnologies	sc-11198
VCP	Western blot	NA	Abcam	ab-11433
Goat anti-mouse	Western blot	IRDye800CW	LiCOR	926-32210
Donkey anti-goat	Western blot	IRDye800CW	LiCOR	926-32214
Goat anti-rabbit	Western blot	IRDye800CW	LiCOR	926-32211
FLI-1	ChIP-Seq	NA	Santa Cruz Biotechnologies	sc-356
Rabbit IgG	ChIP-qPCR	NA	Santa Cruz Biotechnologies	sc-2027
Goat IgG	ChIP-qPCR	NA	Santa Cruz Biotechnologies	sc-2028

Table S2 : List of primers (Related to Figure 1, 2, 3 and 4)

**Primers for RT-qPCR**		
**Genes**	**Forward**	**Reverse**
**Actb**	AGCACAGCTTCTTTGCAG	GATGGAGGGGAATACAGC
**Fli-1**	GTCAATGTCAAGCGGGAGTA	ATGACTCTCCGTTCGTTGGT
**Lmo2**	GAAAGGAAGAGCCTGGAC	ATGGCTTTCAGGAAGTAGC
**Gp1ba**	TAGAGAGAAGGACCGAGTCA	GCTGGTCACTTTGGAGATAC
**Cd41**	CTGCTGACCCTGCTAGTTT	CCCTCTGCTGTCACTCTTC
**Thbs1**	CTCTGCTTTCACAATGGAGT	TTTTGCAGATGGTAACCGA
**Selp**	TGGAATGATGAACCCTGTTT	CAATGGTCTCGATGCACT
**Rab27b**	ATCCCAAATTCATCACCACA	ACCTTAAACGCTTTTCCTGA
**vWF**	TGCAGTTATCTCCTGGCT	AAACTCCCCAAGATACACAG
**Meis1**	ACGCTTTTTGTGACGCTTTT	TCCACTCGTTCAGGAGGAAC
**Trp53**	TGTCATCTTTTGTCCCTTCT	CTTATTGAGGGGAGGAGAGT
**Pdcd1lg2**	GGCCATAGTGATAATCCAGA	GAAGTCTCTTGAGGGTTTCC
**Stat1**	ACAGAAGGAGCTGGACAGTA	AAAAACATCTTGTGGAGCAG
**Tgfb1**	CGAAGCGGACTACTATGCTA	CGAATGTCTGACGTATTGAA
**Primers for ChIP-qPCR**		
**Region**	**Forward**	**Reverse**
**β-Amylase**	CTCCTTGTACGGGTTGGT	AATGATGTGCACAGCTGAA
**Gp1ba pro**	TCACAGGAGCTGATTATCAG	AGACAGACAGTCCTTTGGAG
**Cd41 pro**	CTCTTGAATGCTGTGATGTG	AGGAAGTGGGTAAATGTCCT
**Tgfb1**	GGTGTCAGTAGCTTCTCCAG	GAAATGGGGTGACATAGAGA

**Meis1 +94**	CTGGTGGCAAGAGTTACTTC	CCACTTGACTTTCTCCACA
**Meis1 +55**	GTTGAGGTTTAGGCACTCTG	AGAGGTTCTCACAGGAACAA
**Meis1 +48**	CACTGAGATAGGAACCTGGA	GCATTTCTTGTCACTTCCTC
**Stat1 +31**	CACACCCACTAGGACAAGTT	GTGTTATCACTCAGGCAGGT
**Trp53 +0.9**	GATTCTAGGCTGGTTCTGTG	TAAAACCGGATACTCGGTAA
**E2f2 +5.7**	AGAGTGTTTGCACTGTTTCC	GTGAGAGGCAAATAATCAGG
**Dusp1 +5.5**	AAAGGGAAACTCCTCAGTGT	GTCCGTGGTCTCAACTTAAC
**Pdcd1lg2 +8**	GATCAGGCTTTATTGCTCAC	TCTAGGCTCTTCAGTCTCCA
**Primers for screening Meis1 CRISPR clones**		
**Viewpoint**	**Forward**	**Reverse**
Meis1 ∆94	ATGTCTCAAAACAAACAAAAGC	TGGAGTCACCTTGGGATT
Meis1 ∆55	TCTGACTCACAATTGAAAAAGG	GCACAGAGCGTGTAGACG
Meis1 ∆48	CCCATCATGTAGCATTGTG	AGAGCAATGGAGATATTTGC
**gRNA for deleting Meis1 enhancer**		
	Sense	Antisense
5' Meis1 ∆94	GGTCTACAATAGCTCCTTCC	CGGAAGGAGCTATTGTAGAC
3' Meis1 ∆94	TGAGGGGTTAATGTTAGAGG	CCTCTAACATTAACCCCTCA
5' Meis1 ∆55	GCTATCTCACCCGACCCCCC	GGGGGGTCGGGTGAGATAGA
3' Meis1 ∆55	GCTGAGGAGGGTCGAGCCCT	AGGGCTCGACCCTCCTCAGC
5' Meis1 ∆48	ACTCAGGGCTGGCCGTTGGC	GCCAACGGCCAGCCCTGAGT
3' Meis1 ∆48	ACCTGCTGCTTTAGGTATTC	GAATACCTAAAGCAGCAGGT

TRANSPARENT METHODS

Mice

Ethical approval was obtained from the Committee on the Ethics of Animal Experiments (DEC) of the Erasmus MC as well as from the was obtained from the Ministère Délégué de la Recherche et des Nouvelles Technologies, agreement no. 4936; Direction des Services Vétérinaires, agreement n°69266317 and 7462.
All animal experiments were carried out according to institutional and national and EU guidelines.

Megakaryocyte differentiation, staining and transduction

Fetal liver cells were isolated from E14.5 embryos and homogenized to obtain a single-cell suspension. cKit $(CD117)^+$ cells were isolated by incubating the cells with biotin-conjugated CD117 antibody for 30 minutes followed by 30 minutes incubation with magnetic streptavidin microbeads (Miltenyi biotech) in a buffer containing PBS, 2 mM EDTA and 0.5 % biotin-free bovine serum albumin (Thermo Fisher). Cells were passed in a LS column (Miltenyi biotech) attached to a magnet, washed 3 times with a buffer containing PBS and 2 mM EDTA and the $cKit^+$ cells were collected by adding the plunger to the column. The positive population was then transduced with 20 µL of lentivirus in presence of 8 µg/mL polybrene and cultured for 5 days in IMDM (Life technologies) supplemented with 10 % FCS, 50 ng/mL mTPO, 10 ng/mL mSCF. Cells were then washed 5 times with IMDM, transduced with lentiviruses as above and cultured for 4 additional days in IMDM supplemented with 10 % FCS, 50 ng/mL mTPO.

Constructs

gRNAs targeting the *Meis1* enhancers (sequences in Table S1) were cloned in the BbsI site of the pX459 plasmid (gift from Feng Zhang Addgene plasmid # 62988) (Ran et al., 2013).

Isolation of MkP from WT and FLI-1 KO mice

MkP were isolated from the bone marrow of WT and FLI-1 KO mice following the protocol described by Pronk *et al.* and using a FACS Aria III (Pronk et al., 2007; Starck et al., 2010). The antibodies used are listed in Table S2.

Western blot

Western blot experiments were performed as described previously (Giraud et al., 2014). The primary and secondary antibodies used are listed in Table S1.

Chromatin immunoprecipitation (ChIP)

ChIP experiments were performed as described previously(Giraud et al., 2014). The antibodies used are listed in Table S1.

cDNA synthesis

500 ng of RNA isolated using the TRI Reagent (Sigma-Aldrich) was retro-transcribed into cDNA by using the SuperScript II First Strand Synthesis System (Life technologies) and oligo-dT primers (Life technologies) according to manufacturer's instruction.

Real-time PCR

Real-time PCR was performed using the platinum Taq Polymerase (Life technologies), SYBR green (Biorad) and the primers listed in Table S2 on a Biorad CFX96 apparatus. The specificity and the linear efficiency of each primer have been tested using different amount of mouse genomic DNA or mouse cDNA. Melting curves were checked to confirm the amplification of only the expected amplicon. A negative control was performed in parallel with the real experiment. For gene expression, qPCR reactions were performed in technical duplicates from 5 ng of cDNA and relative DNA levels were calculated using the $\Delta\Delta$Ct method and were normalized to the expression of the *Actb* RNA level. For ChIP, qPCR reactions were performed in technical duplicates from 1/25 of ChIP DNA solution and 1/67 of Input DNA solution. Relative DNA levels were calculated using the $\Delta\Delta$Ct method and were normalized to the Input level.

T2C

T2C experiments were performed as described(Kolovos et al., 2014, 2018) using HindIII as a first enzyme and DpnII as second enzyme. The visualization of the interactome of specific DNA fragments (viewpoints) was performed as described before(Kolovos et al., 2014, 2018).

3C-Seq
3C-Seq experiments were performed as described by Stadhouders *et al.* by using HindIII as first enzyme and NlaIII (*Fut8* enhancer as a viewpoint) or DpnII (*Fut8* promoter as a viewpoint) as second enzyme (Stadhouders et al., 2013). The primers used are listed in Table S1. The samples were sequenced in Illumina Hiseq2500. Raw data were mapped to the reference genome (mm9) and analyzed using the r3Cseq (Thongjuea et al., 2013).

Data availability
The accession number for the FLI-1 ChIP-Seq, the RNA-Seq and the T2C experiments reported in this paper is SRA: SRP158024.

Cell culture and treatment
MEL and HEK cells were cultured in DMEM medium (Lonza) supplemented with 10 % fetal calf serum and penicillin/streptomycin. Cells were induced with 2 % DMSO for 2 days. Expression of control shRNA or shRNAs against Fli-1 mRNA was induced by treating the cells with 100 ng/mL doxycyclin 2 days prior to DMSO treatment. L8057 cells were cultured in vol/vol IMDM/RPMI1640 (Life technologies) supplemented with10 % fetal calf serum and penicillin/streptomycin.

Lentivirus production
20 µg of pLKO.1 (containing the shRNA), 15 µg PAX2 and 5 µg VSV-G plasmids were incubated for 20 minutes with 100 µg PEI and applied to HEK cells. Cells were cultured for 4 hours in DMEM supplemented with 1 % FCS and then for 3 days in DMEM supplemented with 10 % FCS and penicillin/streptomycin. Each day, the medium containing the lentiviral particles was collected and stored at 4 °C. The collected medium was then centrifuged 5 minutes at 1500 rpm, passed through a 0.45 µm filter and ultra-centrifuged 2:15 hours at 20000 rpm at 4 °C. Supernatant was discarded and the lentivirus pellet was resuspended in 100 µL cold PBS. 20 µL were used to transduce cells in presence of 8 µg/mL polybrene.

ChIP in transduced fetal liver cells
Fetal liver cells were isolated from E14.5 embryos and homogenized to obtain a single-cell suspension. Cells were then transduced with 20 µL of lentivirus in presence of 8 µg/mL polybrene and cultured for 3 days in IMDM (Life technologies) supplemented with 10 % FCS, 50 ng/mL mTPO, 10 ng/mL mSCF. Cells were then crosslinked using 1 % formaldehyde and quenched with 0.125 M Glycine. Cells were lysed in a freshly made TE/NP40 buffer (10 mM Tris pH 8, 1 mM EDTA, 0.5 % NP-40, 1 X PIC (Roche Diagnostics)) for 10 minutes in ice. After a centrifugation of 5 minutes at 4 ˚C and 2500 g, the nuclei pellet was resuspend in the NEB buffer (50 mM Tris pH 8, 1 % SDS, 10 mM EDTA, 1 X PIC) and incubated for 10 minutes in ice. The chromatin was then sheared in the Bioruptor (Diagenode) during 30 minutes with the cycle 30 sec ON 30 sec OFF at high intensity. The sonicated chromatin was then precleared using Protein A/G coated agarose beads in the RIPA-150 buffer (50 mM Tris pH8, 0.15 M NaCl, 1 mM EDTA, 0.1 % SDS, 1 % Triton-X100, 0.1 % Na DOC) supplemented with 1 X PIC and 1 % BSA for 3 h at 4 ˚C. Beads were then discarded, new Protein A/G agarose beads attached to 5 µg of specific IgG were added to the pre-cleared chromatin and the mixture was incubated overnight at 4 ˚C. Beads were then washed once with RIPA-150 buffer, twice with RIPA-500 buffer (50 mM Tris pH8, 0.5 M NaCl, 1 mM EDTA, 0.1 % SDS, 1 % Triton-X100, 0.1 % Na DOC), twice with RIPA-$LiCl_2$ buffer (50 mM Tris pH 8, 1 mM EDTA, 1 % NP-40, 0.7 % Na DOC, 0.5 M $LiCl_2$) and twice with TE buffer (10 mM Tris pH 8, 1 mM EDTA). Chromatin was then eluted in Elution Buffer (1% SDS, 0.1 M $NaHCO_3$) and decrosslinked overnight at 65 ˚C. Proteins were then degraded by 20 µg Proteinase K for 1 h at 56 ˚C and chromatin was purified using Phenol:Chloroform, precipitated with 1 volume of isopropanol and washed once with 70 % EtOH.

ChIP-Seq
The samples were sequenced at Illumina Hiseq2500. Raw read files were aligned to the mouse genome (NCBI build 37; mm9) using Bowtie (Langmead et al., 2009) and discarding reads mapping to multiple genomic locations. Data processing and analysis were mainly performed as previously described (Soler et al., 2011 ; Stadhouders et al., 2015 ; Kolovos et al., 2016). The ShortRead package (Morgan et al., 2009) was used to convert these into genome-wide coverage files. Using negative binomial distributions, we assigned P-values and false discovery rates (FDR) to each binding region (Rozowsky et al., 2009), and the final peak lists were compiled according with the following criteria: ≥20 read counts at each peak summit and FDR ≤0.001 (using non-specific, IgG, ChIP-seq data as background). Overlaps between binding peaks in the different datasets were identified using

the “findOverlaps” function from the GenomicRanges suite, while the iRanges package (Lawrence et al., 2013) was used in custom R scripts for annotating the genomic location of peaks and defining the closest gene to each. For motif analysis, +/- 100 bp from the centre of the peaks were selected using the rGADEM Bioconductor package (Droit et al., 2010), which then compares discovered sequences to the Jaspar database (Khan et al., 2018) based on similarity score with TOMTOM (Gupta et al., 2007).
FLI-1, GATA1 and TAL1 ChIP-seq in megakaryocytes were acquired from the ENCODE database (Pimkin et al., 2014; Yue et al., 2014) and analyzed as previously described.The coordinates of the FLI-1 total binding regions and common binding regions in MEL cells and in Megakaryocytes are listed in **Table S3**.

RNA-Seq

RNA were isolated with either the miRNeasy Mini Kit (QIAGEN) (for MEL cells) or miRNeasy MicroKit (QIAGEN) (for MkP) according to manufacturer’s instructions. The samples were sequenced at Illumina Hiseq2500. Reads were aligned to the mouse genome (NCBI build 37; mm9) using Tophat (Trapnell et al., 2009) and default parameters (“no-coverage-search”, “segment-length 18” as input options) while reads that did not map uniquely were discarded. Uniquely mapped reads were counted per RefSeq gene exon using HTseq (Anders et al., 2015) and statistical analysis of differentially-expressed genes was performed via DESeq (Anders and Huber, 2010). In all cases up- and downregulated genes were selected to have at least ±0.6 log2 fold-change in RNA levels. GO analysis was performed by PANTHER (Mi et al., 2019). The list of mis-regulated genes in MEL cells and in MkP after FLI-1 repression are listed in **Table S4**.

SUPPLEMENTAL REFERENCES

Anders S, Huber W. 2010. Differential expression analysis for sequence count data. Genome Biol 11:R106.

Anders S, Pyl PT, Huber W. HTSeq--a Python framework to work with high-throughput sequencing data. Bioinformatics. 2015 Jan 15;31(2):166-9

Droit A, Gottardo R, Robertson G, Li L (2018). rGADEM: de novo motif discovery. R package version 2.30.0.

Gupta S, Stamatoyannopoulos JA, Bailey TL, Noble W. 2007. Quantifying similarity between motifs. Genome Biology 8:R24.

Khan A, Fornes O, Stigliani A, Gheorghe M, Castro-Mondragon JA, van der Lee R, Bessy A, Chèneby J, Kulkarni SR, Tan G, Baranasic D, Arenillas DJ, Sandelin A, Vandepoele K, Lenhard B, Ballester B, Wasserman WW, Parcy F, Mathelier A. JASPAR 2018: update of the open-access database of transcription factor binding profiles and its web framework. Nucleic Acids Res. 2018 Jan 4;46(D1):D260-D266.

Lawrence M, Huber W, Pagès H, Aboyoun P, Carlson M, Gentleman R, Morgan MT, Carey VJ. 2013. Software for Computing and Annotating Genomic Ranges. PLoS Computational Biology 9:e1003118.

Mi H, Muruganujan A, Ebert D, Huang X, Thomas PD. 2019. PANTHER version 14: more genomes, a new PANTHER GO-slim and improvements in enrichment analysis tools. Nucleic Acids Research 47:D419–D426.

Morgan M, Anders S, Lawrence M, Aboyoun P, Pages H, Gentleman R. 2009. ShortRead: a bioconductor package for input, quality assessment and exploration of highthroughput sequence data. Bioinformatics 25:2607–2608.

Rozowsky J, Euskirchen G, Auerbach RK, Zhang ZD, Gibson T, Bjornson R, Carriero N, Snyder M, Gerstein MB. 2009. PeakSeq enables systematic scoring of ChIP-seq experiments relative to controls. Nature Biotechnology 27:66–75.

Soler E, Andrieu-Soler C, Boer E, Bryne JC, Thongjuea S, Rijkers E, Demmers J, van IW, Grosveld F. 2011. A systems approach to analyze transcription factors in mammalian cells. *Methods* **53**: 151-162.

Stadhouders R, Cico A, Stephen T, Thongjuea S, Kolovos P, Baymaz HI, Yu X, Demmers J, Bezstarosti K, Maas A et al. 2015. Control of developmentally primed erythroid genes by combinatorial co-repressor actions. *Nat Commun* **6**: 8893.

Thongjuea, S., Stadhouders, R., Grosveld, F.G., Soler, E., and Lenhard, B. (2013). r3Cseq: an R/Bioconductor package for the discovery of long-range genomic interactions from chromosome conformation capture and next-generation sequencing data. Nucleic Acids Res. *41*, e132**.**

Trapnell C, Pachter L, Salzberg SL. 2009. TopHat: discovering splice junctions with RNASeq. Bioinformatics 25:1105–1111.

Zhang Y, Liu T, Meyer CA, Eeckhoute J, Johnson DS, Bernstein BE, Nussbaum C, Myers RM, Brown M, Li W, Liu XS. 2008. Model-based Analysis of ChIP-Seq (MACS). Genome Biology 9:R137.
